# Navigating SNAP waiver submissions: an Iowa case study

**DOI:** 10.3389/fnut.2026.1771849

**Published:** 2026-03-10

**Authors:** Lyndi Buckingham-Schutt, Rebecca Bucklin, Brandi Janssen, Joelle Johnson, Natoshia Askelson

**Affiliations:** 1 Department of Food Science and Human Nutrition, Iowa State University, Ames, IA, United States; 2 Department of Community and Behavioral Health, College of Public Health, University of Iowa, Iowa City, IA, United States; 3 Department of Occupational and Environmental Health, College of Public Health, University of Iowa, Iowa City, IA, United States; 4 Center for Science in the Public Interest, Washington, DC, United States

**Keywords:** supplemental nutrition assistance program, nutrition incentives, SNAP restrictions, SNAP waiver, policy, policy development, partnership development

## Abstract

This case study describes the process to assess the need, gather information, build partnerships, and develop an evaluation for a Supplemental Nutrition Assistance Program (SNAP) waiver application to the United States Department of Agriculture (USDA) that would have allowed the state of Iowa to test healthy food incentives paired with disincentives. These waivers to test SNAP purchase restrictions and disincentives have been proposed at the national level and have received support from multiple states across the country. Following the rejection of a request to Iowa Health and Human Services (Iowa HHS) to submit a waiver to USDA in November 2024, we conducted a *post-hoc* evaluation of our efforts to develop the proposed waiver with a focus on building strategic partnerships with state officials and other essential partners. We will present the steps taken by the research team to build partnerships with state officials (including challenges experienced with maintaining these partnerships) and the other types of partners who were recruited to move the waiver application forward. We also describe the hurdles that we were unable to overcome and why these hurdles remain despite our efforts. The successes, challenges, and pitfalls of this project offer insights for investigators and state planners hoping to submit similar waiver applications to the USDA.

## Introduction

The Supplemental Nutrition Assistance Program (SNAP), the largest federal nutrition program in the United States, has been administered since the 1960s by the US Department of Agriculture (USDA) to alleviate hunger and malnutrition by increasing low-income households' food purchasing power (1, 2). The USDA administers SNAP, but states and territories are responsible for certifying household eligibility and issuing benefits. A formula is used to calculate the maximum SNAP benefit allotment based on household size and resource and income limits (3). Households are expected to spend ~30% of their own resources on food. Benefits can only be used to purchase staple foods and beverages. They cannot be used to buy alcoholic beverages, tobacco, non-food items, vitamins and supplements, or hot prepared foods (4). Although SNAP is federally authorized and funded, substantial state-level discretion in policy design, administration, and implementation has resulted in wide variation in SNAP participation and outcomes across states, creating opportunities for states to experiment with policy innovations that may improve program reach and effectiveness (5, 6).

Public health experts assert that SNAP offers an opportunity to address long-standing inequities in public health outcomes, particularly the prevalence in diet-related chronic diseases among populations with low-incomes (7–11). However, a disconnect between SNAP as a federal nutrition program and the overall diet quality of its participants has sparked debate over SNAP benefit coverage, with some groups advocating for restrictions on purchases of “unhealthy” foods with SNAP benefits (12–16). This call for purchase restrictions is based on evidence that while SNAP improves household food security (17–19) there is mixed evidence on whether SNAP participation improves diet quality (20–22).

Many politicians, researchers, anti-hunger advocates, industry lobbyists, and public health and nutrition experts are interested in the potential impacts of SNAP purchase restrictions (5, 6, 9, 13, 20, 23–25). “Purchase restrictions” in this article and most commonly in the popular discourse and research literature refer to restricting the purchase of sugar-sweetened beverages (SSBs) and, more recently, sweetened beverages, candy, and prepared desserts, with SNAP benefits. Both lobbyists and anti-hunger advocates oppose purchase restriction strategies, the former being fearful of declining profits and the latter citing SNAP participants‘ autonomy, dignity, and right to food (12), as well as increased stigma in an already stigmatized program (13, 14, 16, 26). At the same time, some public health experts, healthcare providers, and nutrition scientists question whether purchase restrictions will actually improve diet quality and reduce food insecurity among SNAP participants (20). Current evidence is inconclusive, with significant limitations in the generalizability of findings due to methodologic challenges (20, 27, 28); the only randomized controlled trials testing the efficacy of purchase restrictions have been based on income-eligible non-participants' responses, not SNAP participants (27, 28).

Further research to provide conclusive evidence for the impact of purchase restrictions with actual SNAP participants is not possible without USDA approval. Testing changes to SNAP in a real-world setting with SNAP participants, requires a USDA waiver (29). Such waivers, which allow states to temporarily change aspects of the federal program requirements, have been used to increase flexibility, improve program delivery, and meet urgent needs (during COVID-19, federal waivers were highly visible) (30). Typically, a state SNAP agency submits a waiver application to USDA. Prior to 2025, USDA has shown little interest in pilot tests or policy simulations related to purchase restrictions and has denied numerous waiver applications for SNAP pilot projects. However, toward the end of the Biden Administration, USDA indicated an interest in supporting such research projects.

In 2019, our group in Iowa, with support from the Center for Science in the Public Interest (CSPI), started the process for submitting a waiver application that would allow us to explore SNAP-related innovations in Iowa, assess Iowa interest-holders' acceptance and feasibility of various approaches, design a pilot research project, and evaluate pilot implementation. This case study describes our process of assessing feasibility, gathering evidence, building partnerships, and developing a plan to evaluate the impact of an opt-in financial incentive/disincentive (i.e., financial incentives earned by purchasing produce and not purchasing SSBs) intervention on the diet quality of SNAP participants.

## Background

### SNAP pilots and waiver submissions

Historically, SNAP strategies to improve diet quality among participants have had bipartisan support (31). For example, the Food, Nutrition and Conservation Act of 2008 (also known as the 2008 Farm Bill) authorized $20 million for the Healthy Incentive Pilot (HIP) (32), which evaluated the efficacy of financial incentives to increase the purchase of fruits and vegetables. Specifically, the intervention provided SNAP participants with an incentive of $0.30 for every dollar of SNAP benefits spent on targeted fruits and vegetables, allowing households to earn up to $60 per household per month in addition to their regular monthly benefit. HIP led to increased purchase and consumption of a wider variety of fruits and vegetables among SNAP participants, with more meeting federal dietary guidelines and achieving higher overall diet quality; however, intake of non-nutrient foods like sugar sweetened beverages (SSB) did not change (33). Due to HIP's success, the pilot was expanded in the 2014 Farm Bill (34), with further growth in the 2018 Farm Bill including funding dedicated to program evaluation (35).

This systematic study and implementation of healthy eating incentives have led to several program changes. However, evidence that would support adding SNAP restrictions is still limited (36), which is one reason that proposed purchase restrictions have not received USDA approval. Meanwhile, between 2010 and 2024 the USDA has denied four applications (from New York City, Maine, Minnesota, and Massachusetts) to study the effect of restricting SSB purchases with SNAP benefits.

### Why Iowa?

Since 2016, a growing number of national organizations and taskforces have been calling for SNAP to support strategies that would increase intake of nutrient-dense food and limit intake of less nutritious foods (8, 31, 37). In 2018, CSPI–a non-profit organization based in Washinton, DC that advocates for evidence-based and community-informed policies on nutrition, food safety, and health–began funding state organizations to develop recommendations for SNAP healthy eating pilot approaches.

CSPI had a long-standing relationship with Senator Tom Harkin of Iowa. While in office, the senator had a reputation for crafting innovative public health policies, including the Healthy, Hunger-Free Kids Act of 2010, and he was also known for his ability to work “across the aisle” to achieve consensus. In 2021, CSPI partnered with The Harkin Institute for Public Policy and Citizen Engagement (THI)—a policy institute at Drake University in Des Moines, Iowa—to support development of a USDA waiver application.

Iowa's political landscape in 2021 made it an interesting setting for policy innovation. The Republican Party controlled most statewide offices, as well as the state house of representatives and senate, and all federal congressional delegation. Prominent Iowans held leadership roles in the USDA (including the secretary position, held by a former Iowa, Democratic Governor). State agencies and legislators were interested in modifying nutrition-related policies, including restricting the purchase of unhealthy food. Iowa had high rates of obesity (8, 38, 39) and diet-related chronic diseases (40) and low rates of fruit and vegetable consumption (40).

**Figure 1:**
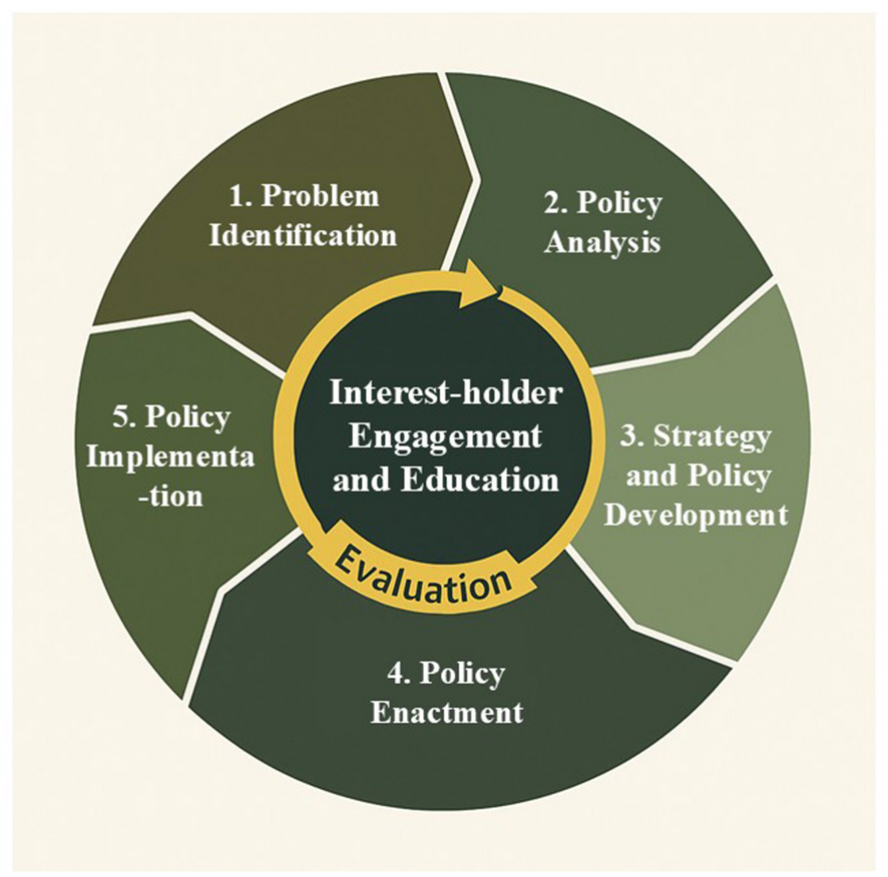
Iowa healthy SNAP waiver development process. This figure illustrates the four phases of the collaborative effort to develop a supplemental nutrition assistance program (SNAP) waiver in Iowa.

State universities and public health researchers collaborated with THI from the beginning on needs assessments, research design, and evaluation planning. CSPI funding supported development of pilot research and an evaluation plan to test an approach that paired incentives and disincentives (Figure 1).

## Project development

**Figure 2:**
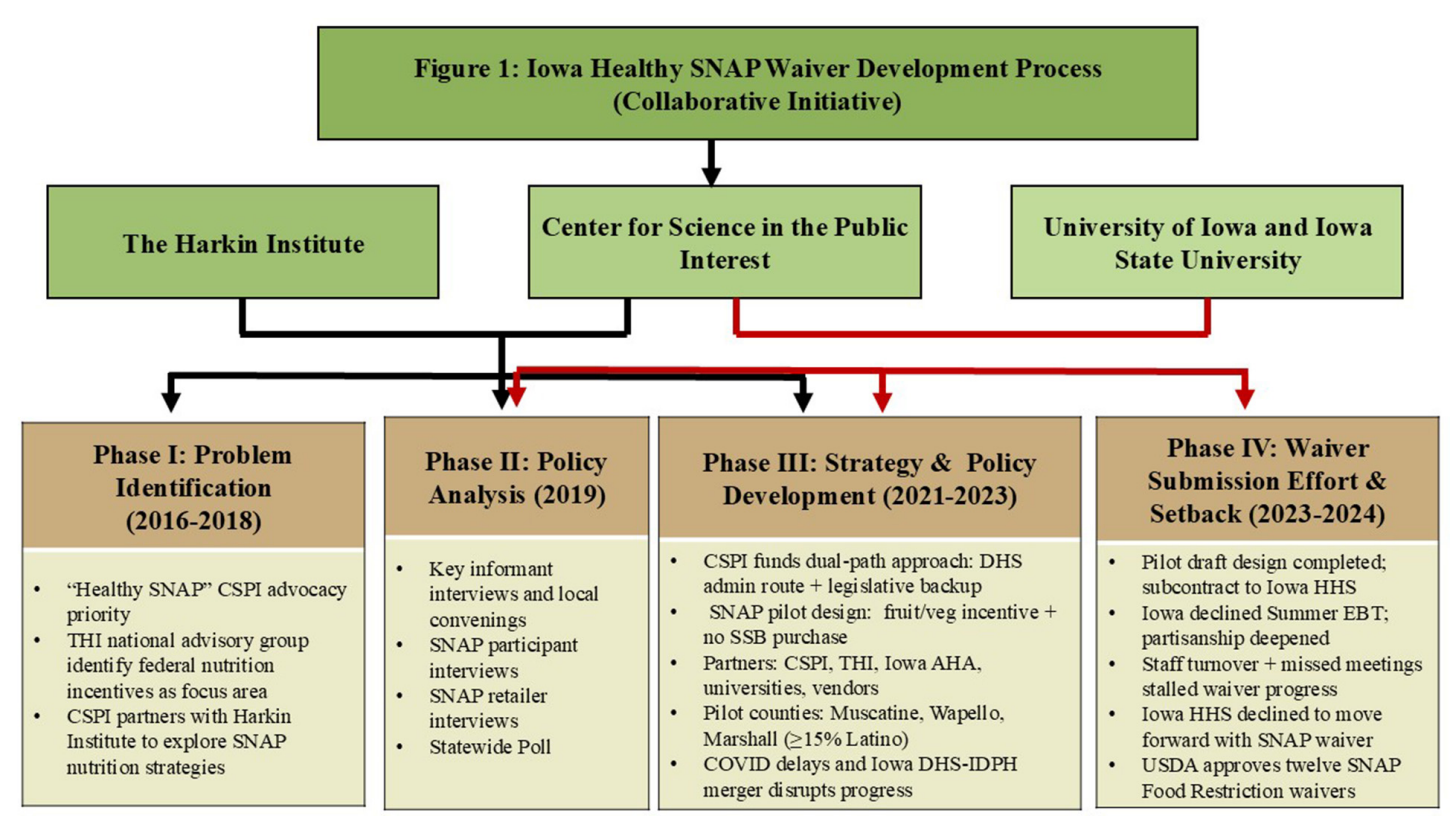
Causal loop diagram mapping the phases to the CDC policy process. This diagram illustrates the five phases of the CDC policy process—problem identification, policy analysis, strategy and policy development, policy enactment, and policy implementation—along with the central role of interest-holder engagement and education and the continuous evaluation component. Adapted from the CDC policy process framework, source: CDC, materials developed by the CDC and are part of the Public Domain.

The first three steps in the Centers for Disease Control and Prevention's Policy Process (41) provide an appropriate framework to describe our development process (and guide future waiver development plans; Figure 2). Our process, in line with the CDC framework, involved three phases: 1) problem identification based on current literature and public perception of the problem, 2) policy analysis, including findings from a report that we compiled (“Strategies to Improve Healthy Eating in SNAP: an Iowa Perspective”) (38), and 3) strategy and policy development that led to a pilot study design.

### Problem identification (phase 1)

Phase 1 of the policy process involves identifying the problem and its root cause. The problem, the prevalence in diet-related chronic diseases among low-income populations, is complex with no single solution. CSPI advocates for evidence-based nutrition and health policy solutions, including opportunities to strengthen SNAP so that it better supports participants ability to access and afford a healthy diet (42). CSPI reviewed and assessed the available research related to SNAP and public health and created a report outlining research on strategies that could strengthen the nutritional and broader health impacts of SNAP (43). Concurrently, in 2018 THI and a national advisory group that included health, wellness, and public health leaders, identified federal nutrition incentives as a key focus area for THI nutrition policy agenda. THI staff conducted a literature review and environmental scan of Iowa data, trends, and events relevant to the broader problem and potential solutions.

In 2018, Senator Harkin introduced THI staff and CSPI staff to foster collaboration on nutrition policy issues. THI and CSPI's shared interest in addressing drivers of diet-related chronic conditions and preliminary work in this area made it easier to collectively move to phase 2 of the policy process.

### Policy analysis (phase 2)

Phase 2 activities took place between June and December of 2019. In this phase, we refined potential approaches and policy options in preparation for Phase 3 and built consensus for strategies that support food and nutrition security within SNAP (20, 37, 38).

THI and CSPI staff identified and engaged diverse interest-holders, including anti-hunger groups, public health, government, academia, community organizations, SNAP participants and retailers, and the public. We explored their perceptions and support of various SNAP strategies, including expansion of financial incentives, use of purchase restrictions, and an opt-in program of paired incentives and disincentives. A full description of our findings was shared in 2020 (38). Figure 2 summarizes the level of support for each approach by interest-holder group and briefly describes interest-holder findings for each group.

#### Interviews and local convenings

We wanted input of community organizations with expertise in public health, nutrition, and food insecurity, as well as SNAP participants. These key informants were identified through various channels, including an advisory group (created to guide the consensus building process) and national, state, and local partners. Participants in structured interviews (*n* = 13) and local meetings (*n* = 38) represented organizations from urban, suburban, and rural settings. The interviews and three convenings (in an urban, micropolitan, and rural communities) were conducted to generate ideas and build consensus around specific pilot approaches to support healthy eating through SNAP.

Analysis of the interviews and convenings revealed strong support for pilot strategies aimed at increasing the accessibility of healthy options, including an opt-in program providing financial incentives for fruit and vegetable purchases in exchange for not purchasing SSBs, expanding nutrition incentive programs to include non-fresh produce and healthy retail marketing strategies. Participants also expressed broader interests: improving dietary quality for all Iowans, increasing wages for low-income individuals, increasing SNAP benefits, and boosting state funding for incentive programs.

#### SNAP participant interviews

Implementation science emphasizes the need to understand the feasibility of a proposed intervention with the actual intervention population. Researchers from University of Iowa assisted with gathering the perspectives and recommendations of SNAP participants. Individual phone interviews were conducted with 37 participants from across Iowa, age 24 to 85 (with an average age of 51). SNAP participants were recruited through flyers and postcards distributed at food banks and pantries (38).

**Figure 3:**
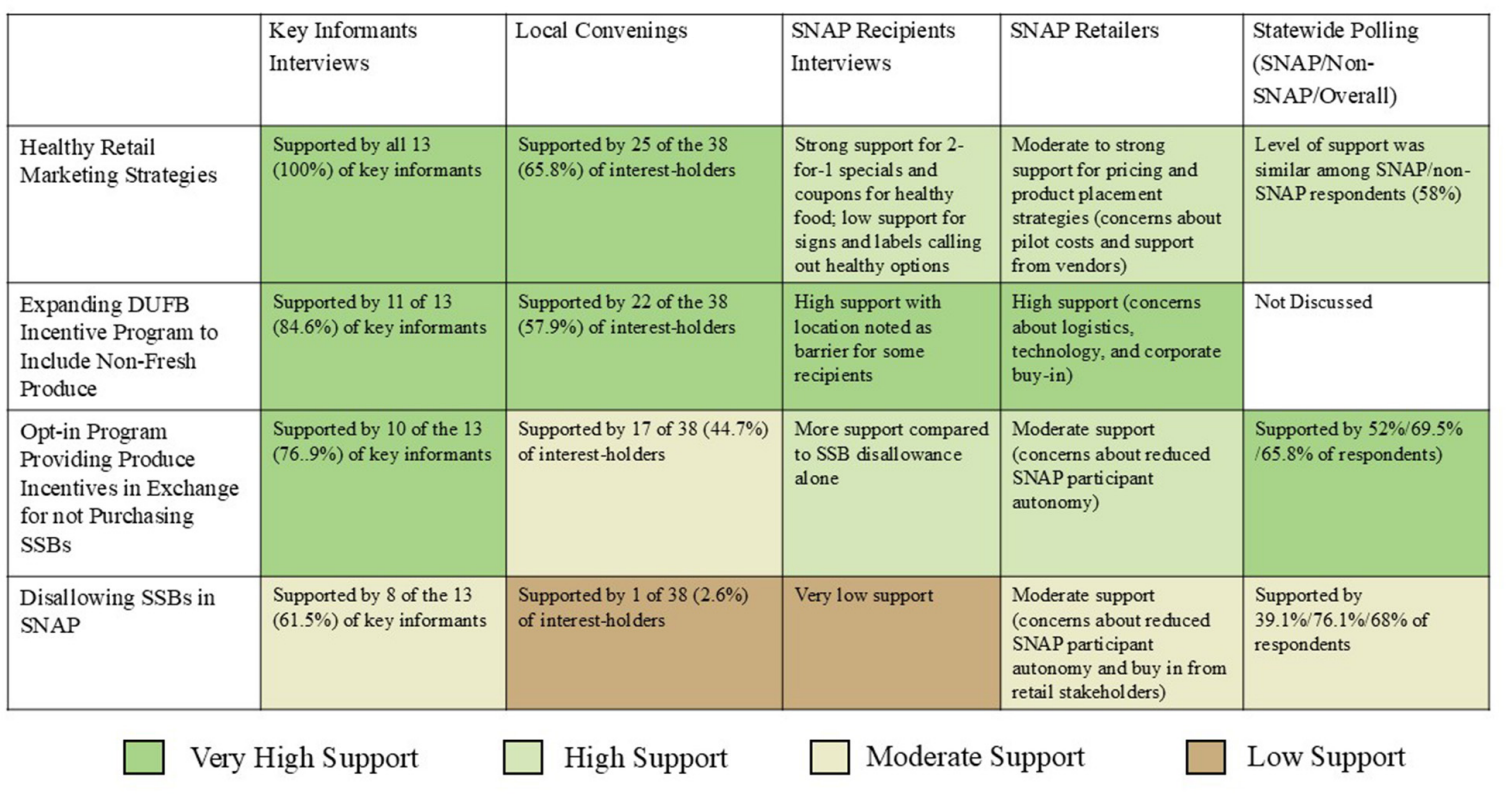
Levels of support for healthy eating strategies across interest-holder groups. This table summarizes stakeholder support for four proposed healthy eating strategies—healthy retail marketing, expanding DUFB incentives, opt-in produce incentives, and disallowing SSBs in SNAP—based on interviews, convenings, retailer input, recipient feedback, and statewide polling, with color coding indicating support levels.

These participants responded positively to initiatives like additional SNAP benefits for fruits and vegetables and dollar-matching incentives (Figure 3). The “Double Up Food Bucks” program, an existing SNAP program that doubles the value of SNAP dollars when spent on fresh produce, was well received, though locations where Double Up Food Bucks could be earned and redeemed (limited to farmers markets and certain retailers) was noted as a barrier. They also supported additional SNAP benefits for other healthy items like frozen fruits and vegetables, whole wheat bread, whole grain products, and milk.

Conversely, a proposed policy restricting the purchase of SSBs received little support, with participants expressing strong negative opinions. They felt it was unfair and could restrict purchasing beverages recommended by doctors. However, a proposed policy where participants would earn additional SNAP benefits for not purchasing sugary drinks was positively received, because they felt that approach would motivate them to reduce SSB consumption.

Conducting interviews with Iowa SNAP participants was critical to understanding the need for proposed policy change and acceptability of a proposed change. Within this group, we found that cost remains a major barrier to purchasing healthy foods, and strategies to increase the amount SNAP benefits that households receive or provide financial incentives for fruits and vegetables could help overcome this barrier.

#### Retailer engagement

In the fall of 2019, THI and Iowa State University interviewed SNAP retailers to develop informed recommendations, and implementation and evaluation plans for a SNAP waiver design that pairs financial incentives for fruits and vegetables with disincentives for purchases of sugar-sweetened beverages (44).

In-person interviews were conducted with food retail personnel at the corporate level (*n* = 2) and local level (*n* = 6). Generally, they viewed financial incentives for fruit and vegetable purchases as a win-win approach, anticipating increased customers and sales. However, they also noted challenges: stigma, consumer privacy, procurement of healthy food items, regulations, and point of sale system technology issues (Figure 3). All emphasized the need for retailer participation in SNAP healthy-eating strategies and education for SNAP participants to ensure effective program functioning.

Retailers raised concerns that restricting SNAP purchases or imposing SSB disincentives could create stigma and limit participant autonomy. While some believed disincentives might boost healthy food sales and promote social responsibility, challenges included retailer buy-in, potential customer loss, and limited participant education and understanding.

Overall, the recommendations from retailers included ensuring that financial incentive and disincentive programs are easy to use and understood by both consumers and retailers, modernizing the current EBT card, and ensuring that programs do not increase stigma.

#### Statewide polling

In December 2019, THI and an Iowa marketing research firm conducted an online survey of 500 adults aged 18 and older to determine whether Iowans supported or opposed healthy eating strategies through SNAP. The survey, developed by CSPI and adapted by THI, was delivered online over a 2-week period. The sampling strategy intentionally ensured that approximately 20% of respondents were SNAP participants, slightly exceeding the proportion of Iowans enrolled in SNAP, to allow for meaningful comparison of perspectives, and respondent demographic characteristics were collected and are reported elsewhere (38). Respondents came from 96 of the 99 Iowa counties. They represented a mix of gender (54% female), age (26% 18–34 and 32% 65 or older), marital status (57% married), education (46% some college or less), and political affiliations (35% Democrat and 35% Republican), and 20% of them were SNAP participants.

Findings showed broad support for financial incentives, with 75% of adults overall and 81% of SNAP participants finding that type of strategy very helpful/helpful. Respondents from all income categories supported providing more money for fresh fruit and vegetable purchasing, with the highest support from people making less than $50,000 (79%). When asked about their opinion on whether SSBs should be an allowable SNAP purchase, 68% of respondents did not think SNAP benefits should be used to purchase SSBs, with higher opposition among Republicans (83%) than Democrats (64%) or Independents (59%). Conversely, 62% of SNAP participant respondents felt that SSBs should be available for purchase with SNAP benefits. An opt-in program that pairs a financial incentive for fresh produce in exchange for not purchasing SSBs was supported by 70% of all respondents, with higher support among Republicans (74%) compared to Democrats (68%) and Independents (56%). Among SNAP beneficiaries, 52% supported this strategy.

Our Phase 2 findings aligned overall with the national dialogue around SNAP purchase restrictions. Most SNAP participants and key interest-holders, including food retailers, did not favor purchase restrictions. Only Iowa Republicans showed high support for purchase restrictions. However, the majority of all survey respondents (SNAP and non-SNAP participants) favored disincentives (70%), but only marginally over outright purchase restrictions (68%). Our analysis also provided insight into how a pilot program would be perceived and potential implications for future policy implementation.

### Strategy and policy development (phase 3)

Our policy analysis phase provided the direction and information we needed to design a pilot for a USDA waiver application. Although the original timeline for Phase 3 was 1 year (March 2021-June 2022) the process took over 3 years and ended with unexpected results.

First, the global COVID-19 pandemic occurred, with its significant challenges to public health infrastructure and workforce capacity. The pandemic had both positive and negative impacts on our process, increasing funding opportunities and attention to food and nutrition security efforts, but changing the political environment in ways that had long-term, severe repercussions on waiver submission success.

#### Partnering with state government and pilot design and evaluation planning (2021–2022)

In early 2021, we identified two possible avenues for state government support of our waiver submission: administrative support (approval from the Iowa Department of Human Services [Iowa DHS] and the Governor) or legislative support (a bill requiring Iowa DHS to submit a waiver). We aimed to work with Iowa DHS and the Governor's office, with a legislative campaign as backup. To avoid delays, we pursued both administrative and legislative strategies simultaneously.

Our team handled communications with state agencies and the Governor's office, and we partnered with the Iowa American Heart Association (AHA) to handle direct advocacy. Lobbying is an important part of the policy process; however, institutes of higher education have restrictions on lobbying and may lack important connections with policymakers and players.

While the designated team members moved ahead with their partnering/lobbying efforts, researchers from THI, University of Iowa, and Iowa State University began to design a pilot research study and evaluation plan. The strategy of our SNAP Incentive Project aimed to offer SNAP participants a $1.00 financial incentive for any $1.00 spent on purchases of fruits and vegetables provided they did not purchase any SSBs using SNAP benefits in the same transaction. SSBs included any non-alcoholic beverage, whether carbonated or non-carbonated, sold for human consumption that contains any added caloric sweetener (45). This paired intervention reflected our Phase 2 findings and the increasing national attention on strategies that paired financial incentives with disincentives.

The research team reviewed the literature on financial incentives and purchase restriction interventions; determined pilot aims, research questions, primary and secondary outcome measures, tools, and timelines; designed the analysis; and chose pilot sites. By August 2021, we were able to share the pilot plan and evaluation design with CSPI staff and external experts. The complex plans were strengthened by ongoing discussions with interest-holders such as grocers, technology partners, and Iowa community partners.

In November 2021 we presented our report and initial pilot design to Iowa DHS. Staff at Iowa DHS had been helpful in our policy process from the early stages along with staff from Iowa Department on Public Health. Iowa DHS was supportive of the waiver application but needed to secure leadership approval before they agreed to submit the pilot waiver. Between late fall 2021 and summer 2022, we continued our regular meetings with the Iowa DHS.

Although we had made significant progress and had overall strong support, changes in the state's political landscape kept us from completing our waiver application within the original March 2021–June 2022 timeframe. In the last half of 2022, we pushed ahead, completing a draft of the aims and research questions, adding a statistician to the research team to support sample size calculation, establishing additional relationships with Iowa food vendors, and selecting three pilot counties (each with a micropolitan community of at least 15% Latino members). We began to engage community partners in these counties, secure partnership agreements, and create specific plans for pilot implementation and evaluation. We also continued meeting with the Iowa DHS, which underwent a departmental reorganization that ultimately affected our ability to complete and submit a SNAP waiver application.

#### Political changes (2022–2024)

Funding extensions from CSPI allowed us to continue working on our waiver submission through 2023. During that time, engagement and communication with the Iowa DHS became less frequent, stalling our progress and indicating either the agency's lack of interest or inability to submit a waiver application. Both explanations were concerning: lack of interest affected submission, and inability foreshadowed implementation challenges.

The biggest factor in their disengagement was the summer 2022 merger of the Iowa DHS and the Iowa Department of Public Health into the newly named Iowa Department of Health and Human Services (Iowa HHS) (46). The new agency's changing staff roles and reassigned positions sowed confusion in our waiver-related partnerships and necessitated a restart of the entire agency approval process. SNAP administration was merged with the former Department of Public Health bureau that oversaw other nutrition-related programs (such as SNAP-ed, WIC).

Despite challenges locally, we were encouraged by growing interest at the federal level. In September 2022, the Biden administration held a White House Conference on Hunger, Nutrition, and Health (47). The first such conference took place in 1969 under President Richard Nixon and led to major advancements in federal nutrition assistance programs. The 2022 conference, which aimed to build on this legacy by addressing current challenges related to hunger and diet-related diseases (37), brought the promise of future funding for pilot programs or new avenues for pilot approval that could support our work in Iowa.

Throughout 2023, we continued developing the waiver application and pilot design. By the end of 2023, our team had completed a draft pilot design and evaluation plan but had been unable to finalize it without consistent collaboration with Iowa HHS. An unexpected occurrence in December 2023 was the Governor's announcement that Iowa would not participate in the 2024 USDA Summer EBT program. She stated, “Federal COVID-era cash benefit programs are not sustainable and don't provide long-term solutions for the issues impacting children and families. An EBT card does nothing to promote nutrition at a time when childhood obesity has become an epidemic” (48). Her justification highlighted state leaders' recognition that poor nutrition among low-income families was a pressing issue, but it deepened the debate over the purpose of federal assistance programs, increased partisan arguments about nutrition policies, and alienated important local interest-holders and partners within Iowa.

In 2024, with our progress stalled, we pivoted our strategy and University of Iowa redirected a portion of their project funding toward a subcontract with Iowa HHS, hoping that supporting dedicated personnel would facilitate approval of the waiver request by ensuring regular communication and collaboration. However, we faced an immediate setback when the main point person for the waiver application process left Iowa HHS. Once a new staff member was oriented in late summer, we managed to schedule biweekly meetings with two other Iowa HHS team members, but the agency had still not officially approved our SNAP waiver submission.

In November 2024, the waiver submission team met with Iowa HHS leadership, who expressed some interest in a waiver proposal aligned with state healthy eating and active living priorities, but at the same time it became apparent they were exploring other programmatic changes and strategies (49, 50). Ultimately, they made it clear they were no longer interested in submitting a waiver application with our proposed pilot design.

At the time of writing this paper, the USDA has approved eighteen state-level SNAP Food Restriction waivers, including one from Iowa, that limit the purchase of certain items such as sweetened beverages and candy (51). These waivers emerged in a shifting political landscape. Following the election of President Trump in 2024, the “Make America Healthy Again” campaign rhetoric called for dismantling perceived corruption in the food industry and reforming federal nutrition programs. This included critiques of SNAP purchases of sugary beverages and candy. In parallel, the administration proposed, and in some cases enacted, significant cuts to SNAP funding.

Waiver requests are publicly available on the USDA SNAP Food Restriction Waivers website along with a summary of their proposed projects (51). U.S. Code mandates that all SNAP pilot projects include “an evaluation component to determine the effects of the project”. The quality and depth of the SNAP Food Restriction waiver evaluation plans vary significantly based on the description included in the original request. For example, Arkansas provides only a single-sentence description (51), while Idaho outlines a multi-page, multi-method evaluation strategy (51). Iowa's plan proposes collaboration with SNAP-Ed, WIC, and Medicaid to collect participant-level outcome data, including health metrics such as BMI and dietary behavior changes.

Of note, we were not involved or consulted in developing the 2025 Iowa waiver. Iowa's proposal heavily differs from our waiver design and evaluation. Unlike the approved Iowa waiver, we designed a pilot randomized controlled trial to test relative efficacy of two SNAP treatment groups (a financial incentive for fruit and vegetable purchases group and a financial incentive for fruit and vegetable purchases in exchange for not purchasing SSBs group) relative to a SNAP control group. The primary outcomes of interest were changes in fruit and vegetable purchasing, consumption behavior, diet quality with secondary outcomes of home food environment and food and nutrition security. Comparatively, the approved evaluation plan was not designed to establish if there is a causal link between treatment effect (SNAP restrictions) and outcomes (“purchase of healthier food items”, “general welfare”, and “health and wellbeing”) (52). All eighteen states technically meet the federal requirement by including an evaluation component; however, the rigor, methodological clarity, feasibility, and ethical considerations are lacking considering the magnitude of proposed program change.

This raises concerns about whether these evaluations can meaningfully assess the waivers' intended outcomes—namely, the Trump Administration's goal to “strengthen and restore nutritional value within SNAP” (51)—or inform future policy decisions using evidence-based information, which was the goal of our proposed waiver submission and evaluation.

## Discussion

Policy decision-making related to SNAP is occurring within a rapidly evolving political environment in which empirical evidence does not consistently guide policy formulation or enactment. Increasingly, both state and federal policymakers are considering legislation that would temporarily restrict SNAP purchases without clear evidence regarding feasibility, acceptability, or effectiveness. Our findings, supported by other studies, show that SNAP participants do not favor these restrictions (53–56).

Throughout our project, we prioritized interest-holder engagement and evaluation planning. These elements are essential components of the CDC policy process framework and emphasized in policy process research as critical to effective policy formulation and learning (57). However, our experience suggests that these practices are not consistently embedded in contemporary policy processes. When policy change occurs without systematic evaluation or engagement from people impacted by the potential policy, uncertainty surrounding policy impacts persists, and opportunities for evidence-informed refinement are limited.

### Lessons learned for future applications

Although our waiver application was ultimately unsuccessful, we gained insight into the impact of an evolving, complex political environment on a policy process. Policy process research highlights that policy formulation is rarely linear and is instead influenced by competing coalitions, institutional and administrative constraints, and shifting political environments (57, 58).

#### Navigating policy challenges

We now understand how crucial it is, when seeking to apply for a SNAP waiver, to be mindful of the obstacles in the policy process such as intergovernmental dynamics, federal-state alignment, agency mandates, electoral cycles, legislative priorities, partisan dynamics, and bureaucratic personnel and leadership. The complexity and public perceptions of the issue may significantly impact the level of difficulty in moving the waiver request forward. In retrospect, a more comprehensive landscape analysis may improve our ability to anticipate and respond to emerging barriers. Such preparation may be particularly important for policy initiatives addressing politically salient and heavily influenced by the social and cultural environment, such as nutrition assistance and benefit design.

#### Essential elements for policy analysis

Consistent with established models of policy formulation and change, our experience underscores the importances of strong relationships and partnerships, sufficient time and funding, input from people impacted by the potential policy, expertise from organizational and research partners and the actual policy makers, and trust in people, partners, and the process (57). Early and frequent engagement of interest-holders and partners, including an advisory group, was key during the policy analysis phase. We spent 3 months collecting the data for our project, after spending more than a year preparing for consensus building. Funding was critical, supporting dedicated staff and compensating contributors which allowed us to pay for others' expertise, time, and in turn, build trust with our partners.

#### Challenges in policy development

Aligning values between advocates and government officials is an important pathway for policy change but recognized as a challenge in policy development (58, 59). Shifts in political leadership, the political environment, agency restructuring, and competing policy agendas can further complicate this alignment (57). Although our proposed work aligned with the broader goals across multiple administrations, these goals were framed differently over time, influencing how SNAP-related innovations were perceived and prioritized. External and internal events, and urgent demands on agency capacity may also limit the ability to make progress (59). Future efforts may benefit from clearer communication of shared objectives and explicit recognition of the constraints inherent in the broader political systems that operate beyond our control.

#### Strengths and limitations

This work required substantial, sustained funding and collaboration among local researchers with expertise in nutrition and policy evaluation, which added credibility and rigor. Such resources and expertise may not be feasible for others. As a case study, findings may not apply broadly but can inform groups navigating SNAP waivers, purchase restrictions, and federal–state collaboration.

## Conclusion

The political environment influenced our effort to conduct novel research on SNAP policy, and the outcome reflects broader structural and institutional factors. Although empirical evidence does not consistently drive policy change, particularly when it conflicts with prevailing political ideology or agenda, strategic adaption within policy frameworks may still enable researchers and interest-holders to navigate and inform the dynamic landscape of public policy.

## Data Availability

The data analyzed in this study is subject to the following licenses/restrictions: The data/dataset can be available upon request to the corresponding author of this paper. Requests to access these datasets should be directed to lbschutt@iastate.edu.
